# A second-look endoscopy may not reduce the bleeding after endoscopic submucosal dissection for gastric epithelial neoplasm

**DOI:** 10.1186/1471-230X-14-152

**Published:** 2014-08-23

**Authors:** Fumiaki Takahashi, Naoto Yoshitake, Takashi Akima, Hitoshi Kino, Masakazu Nakano, Chieko Tsuchida, Kohei Tsuchida, Keiichi Tominaga, Takako Sasai, Hironori Masuyama, Hideyuki Hiraishi

**Affiliations:** 1 Department of Gastroenterology, Dokkyo Medical University, 880, Kitakobayashi, Mibu, Shimotsuga, Tochigi 321-0293, Japan

**Keywords:** Postoperative bleeding, Gastric neoplasm, Endoscopic submucosal dissection, Second-look endoscopy

## Abstract

**Background:**

Gastric endoscopic submucosal dissection (ESD) has gradually come to be recommended as the optimal treatment for early gastric cancer; however, one of the primary issues is postoperative bleeding. Although second-look endoscopy is conventionally performed to reduce the risk of postoperative bleeding, its benefit has not yet been clearly elucidated. The objective of this study was to elucidate the benefit of second-look endoscopy.

**Methods:**

A total of 459 lesions in patients were underwent gastric ESD from May 2004 to April 2013 at our hospital were included in the analysis. The patients were divided into those who had bleeding within 24 hours after ESD (immediate bleeding) and those in whom bleeding occurred 24 hours or more after the procedure (delayed bleeding); the underlying disease, age, lesion site, diameter of the resected specimen, and lesion diameter were analyzed to identify the risk factors for postoperative bleeding after ESD.

**Results:**

Post-ESD immediate or delayed bleeding occurred in 23 of the 459 cases (5.0%). Second-look endoscopy was performed in 210 of 447 cases (47.0%) excluding 12 cases with immediate bleeding; in the remaining 237 of the 447 cases (53.0%), it was not performed. Post-ESD delayed bleeding occurred in 6 of the 210 cases (2.9%) and 5 of the 237 cases (2.1%), with no statistically significant difference between the two groups. Overall, the following factors were identified as the risk factors for postoperative bleeding: young age (*P* = 0.005), lesions in the L segment (*P* = 0.042), and large size of the resected specimen (*P* = 0.005). The risk factors identified in the immediate bleeding group were lesions in the L segment (*P* = 0.032), large size of the resected specimen (*P* < 0.001), and large tumor size (*P* = 0.011), and those in the delayed bleeding group were young age (*P* = 0.013) and concomitant renal disease (*P* = 0.011).

**Conclusions:**

The results of this study suggest that second-look endoscopy after gastric ESD may not be useful for preventing postoperative bleeding.

## Background

Gastric ESD has gradually come to be recommended as the optimal treatment for early gastric cancer. This technique can now be used for the resection of large lesions and ulcer lesions which cannot be resected by traditional endoscopic mucosal resection [1]– [3].

Postoperative bleeding is one among the major complications of ESD. According to past reports, post-ESD bleeding occurs in an estimated approximately 5% of cases [4]– [6]. While the frequency of postoperative bleeding is gradually decreasing owing to the development of post-ESD coagulation therapy and use of proton pump inhibitors (PPI), it remains one of the primary issues that need to be resolved in relation to ESD. Second-look endoscopy after hemostasis for peptic ulcer bleeding has been reported to be useful for the prevention of rebleeding [7]– [9]. Therefore, second-look endoscopy is also conventionally performed post-ESD at many institutions; however, its benefit has not yet been elucidated.

Here we conducted a retrospective study to examine whether second-look endoscopy might be useful for the prevention of post-ESD bleeding. We also evaluated the risk factors for postoperative bleeding.

## Methods

### Patients and lesions

We targeted a total of 488 lesions in patients who underwent gastric ESD between May 2004 and April 2013 at our hospital. In cases with multiple synchronous lesions, those lesions that showed deeper invasion or were larger in diameter if the invasion depth was the same were included. After exclusion of a total of 29 lesions (11 with a residual cancer lesion, 12 with perforation, 2 with aspiration pneumonitis, 1 in which the treatment was switched to open surgery, and 3 in which no evidence of cancer was found in the resected specimen), a total of 459 lesions (405 lesions of early gastric cancer, 54 lesions of gastric adenoma) were considered to be evaluable. Table 1 shows the clinicopathological characteristics of these patients.

**Table 1:** Clinicopathological features of patients and gastric lesions

Age (year, mean ± SD)	71.4 ± 8.8
Gender Man/Woman	344 (74.9%)/115 (25.1%)
Lesion	
Elevated type/Depressed type	256 (55.8%)/203 (44.2%)
Tumor location U/M/L	75 (16.3%)/179 (39.0%)/205 (44.7%)
Size of resected specimen (mm, mean ± SD)	40.3 ± 15.3
Tumor size (mm, mean ± SD)	17.2 ± 11.0
Histopathology Adenoma/Carcinoma	52 (11.3%)/407 (88.7%)
Depth of invasion (M/SM1/SM2)	405 (88.2%)/39 (8.5%)/15 (3.3%)
Ulcerative findings absent/present	426 (92.8%)/33 (7.2%)
Resectability	
One-piece resection	446 (97.2%)
Complete resection	403 (87.8%)
Underlying diseases	
Heart disease	72 (15.7%)
Renal disease	21 (4.6%)
Hepatic disease	45 (9.8%)
Pulmonary disease	36 (7.8%)
Brain disease	45 (9.8%)
Hypertension	209 (45.5%)
Diabetes mellitus	75 (16.3%)
Hyperlipidemia	67 (14.6%)

Prior to the ESD, the patients had undergone endoscopic examinations, including chromoendoscopy, magnified endoscopy, endoscopic ultrasonography, and biopsy, and thoracoabdominal computed tomography. Gastric ESD was indicated for early gastric cancers satisfying the criteria of Gotoda et al., lesions that were strongly suspected as being cancerous, and adenomas for which patients requested resection [10,11].

This study protocol was approved by Dokkyo Medical University Ethics Committee. All patients gave written informed consent before the procedure.

### ESD procedure and management

Patients orally received rabeprazole sodium 20 mg/day from the day prior to ESD to increase the gastric pH and achieve easy hemostasis at the time of ESD [12,13].

At the time of the ESD procedure, the patients received pentazocine 15 mg/dose/h and continuous propofol intravenous infusion for sedation. Propofol was administered in accordance with the method described in the paper of Kiriyama et al. [14] The ESD procedure is described elsewhere [15]– [17]. In short, the peripheries of lesions were marked using the Dual Knife (KD-650 L; Olympus). A local injection solution was prepared by mixing glycerol and sodium hyaluronate at a 1:1 ratio and adding adrenaline and indigo carmine. The solution was locally injected into the submucosal layer [18,19]. Next, the lesion was circumferentially incised with a margin of 5 mm outside the marking using Dual Knife or IT Knife (KD-610 L, Olympus), followed by the resection of the submucosal layer below the lesion, just above the muscle layer. If there was a little bleeding, hemostasis was achieved with the knife used during the ESD procedure. However, in the event of moderate to severe bleeding, hemostasis was achieved using Coagrasper (FD-411QR, Olympus). In most cases, the bleeding could be stopped with hemostatic forceps; however, in rare cases in which the bleeding could not be stopped, the EZ Clip (HX-610-135, Olympus) was used. Immediately after the ESD, the exposed blood vessels at the base of the ulcer were treated with hemostatic forceps or a clip, to the extent possible [19,20].

In general, the patients underwent blood tests and thoracoabdominal radiography on the day after the ESD; if there were no problems, they were allowed to take fluids; from day 2 after ESD, they were allowed to take meals orally, starting with rice gruel. In case second-look endoscopy was performed, it was performed within a few days after the ESD (1.24 ± 0.53 days, range 1–3 days). When the second-look endoscopy revealed hemorrhage or exposed vessels, hemostasis was performed using the hemostatic forceps or a clip. When perforation or post-ESD bleeding was observed, the schedule of discharge and meal resumption was changed depending on the individual patients’ condition. If post-ESD bleeding occurred, emergency endoscopy was performed, and endoscopic hemostasis was performed using clipping or cautery. If the patients had no problems during the course of hospitalization, they were discharged within 1 week after the ESD.

During the interval period from the ESD to resumption of oral meal intake (usually 2 days), the patients were managed by twice-daily intravenous administration of 20 mg omeprazole or 30 mg lansoprazole. After oral intake of meals was resumed, the patients were given oral rabeprazole sodium 20 mg/day for 8 weeks [21].

If the patients were receiving oral anticoagulant or antiplatelet drugs, these drugs were suspended in accordance with the Gastroenterological Endoscopy Guidelines, Version 3 [22]. The drug administrations were resumed from day 2 after the ESD, i.e., at the same time as the resumption of oral meal intake.

Patients were instructed to visit the hospital immediately if they noticed hematemesis or melena after discharge from the hospital.

### Data analysis

Post-ESD bleeding was defined as postoperative hematemesis or melena requiring endoscopic hemostasis. Post-ESD bleeding diagnosed within 24 hours after the ESD was defined as immediate bleeding, while bleeding diagnosed later than that was referred to as delayed bleeding. In order to evaluate the benefit of second-look endoscopy, the frequency of delayed bleeding was examined between patients who underwent/did not undergo second-look endoscopy.

The following factors were analyzed to identify the risk factors for post-ESD bleeding: age, sex, lesion site (upper, middle, lower stomach), lesion form (raised, depressed), size of the resected specimen, lesion size, pathological findings (adenoma, carcinoma), whether an ulcer was formed/not formed, whether en bloc resection was possible or not, the underlying diseases (hypertension, renal, cardiac, pulmonary or brain disease, diabetes, dyslipidemia), and the status of treatment with antiplatelet and anticoagulant agents (yes/no).

### Statistical analysis

Univariate analysis was performed on each item. The age and sizes of the resected specimen and lesion were analyzed by Student’s *t*-test. Chi-square analysis was performed on other data, while Fisher’s exact test was used for the items with an expected value of 5 or more. A *P* < 0.05 was determined as statistically significant.

## Results

Post-ESD bleeding was observed in 23 of the 459 cases (5.0%). Immediate bleeding and delayed bleeding were observed in 12 (52.2%) and 11 cases (47.8%), respectively. Bleeding could be stopped in all patients by endoscopic treatment, and none of the patients required surgical treatment. Postoperative bleeding occurred 8 days or later in 3 patients, and all of them were on treatment with antiplatelet agents.

**Figure 1:**
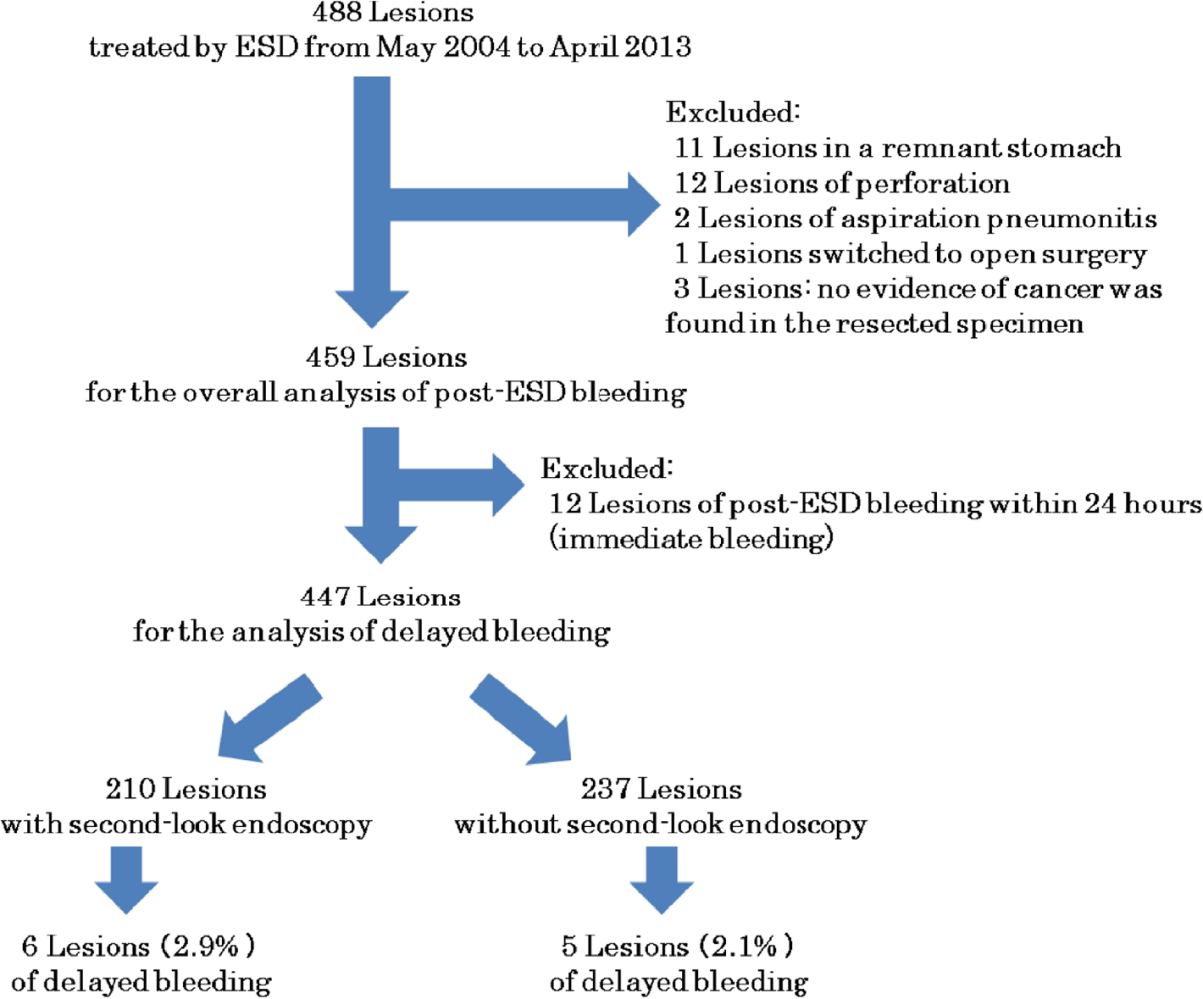
Flowchart showing the analysis of the usefulness of second-look endoscopy to prevent the bleeding after ESD.

Second-look endoscopy was performed in 210 of 447 patients (47.0%) excluding 12 cases with immediate bleeding; and the remaining 237 patients (53.0%) did not undergo second-look endoscopy. No statistically significant differences were observed between the groups that underwent/did not undergo second-look endoscopy in the age, gender ratio, tumor location, macroscopic type, tumor size, histopathology, depth of invasion, or patients’ medical history (Table 2). Postoperative bleeding was observed in 6 of the 210 patients (2.9%) in the endoscopy group and 5 of the 237 patients (2.1%) in the non-endoscopy group, the difference not being statistically significant; i.e., the postoperative bleeding rates were comparable in the two groups (Figure 1).

**Table 2:** Comparison of characteristics between second-look endoscopy group and non-endoscopy group

	**SLE group (n = 210)**	**Non-SLE group (n = 237)**	***P *** **value**
Age (year, mean ± SD)	71.5 ± 8.3	71.6 ± 9.2	0.892 (NS)
Gender Man/Woman	163 (77.6%)/47 (22.4%)	172 (72.6%)/65 (27.4%)	0.263 (NS)
Tumor location			
U	30 (14.3%)	45 (19.0%)	0.315 (NS)
M	79 (37.6%)	97 (40.9%)	0.537 (NS)
L	101 (48.1%)	95 (40.1%)	0.108 (NS)
Macroscopic type			
Elevated type/Depressed type	115 (54.8%)/95 (45.2%)	132 (55.7%)/105 (44.3%)	0.918 (NS)
Tumor size			
(mm, mean ± SD)	16.5 ± 9.0	17.5 ± 11.8	0.309 (NS)
Histopathology			
Adenoma/Carcinoma	30 (14.3%)/180 (85.7%)	41 (17.3%)/196 (82.7%)	0.460 (NS)
Depth of invasion			
M/SM	189 (90.0%)/21 (10.0%)	223 (94.1%)/14 (5.9%)	0.152 (NS)
Heart disease	35 (16.7%)	34 (14.3%)	0.584 (NS)
Renal disease	7 (3.3%)	14 (5.9%)	0.289 (NS)
Hepatic disease	23 (11.0%)	19 (8.0%)	0.368 (NS)
Pulmonary disease	13 (6.2%)	20 (8.4%)	0.661 (NS)
Brain disease	24 (11.4%)	18 (7.6%)	0.221 (NS)
Hypertension	92 (43.8%)	121 (51.1%)	0.126 (NS)
Diabetes mellitus	36 (17.1%)	38 (6.0%)	0.851 (NS)
Hyperlipidemia	23 (11.0%)	41 (17.3%)	0.056 (NS)

The risk factors for post-ESD bleeding were evaluated, including the patients’ medical history. Overall, the risk factors for postoperative bleeding were younger age (66.4 ± 11.3 vs. 71.7 ± 8.6 years, *P* = 0.005), lesions in the L segment (L vs. UM, *P* = 0.042), and large resected specimens (49.1 ± 25.8 vs. 39.9 ± 14.4 mm, *P* = 0.005) (Table 3). Analysis of the immediate bleeding subgroup revealed that postoperative bleeding occurred more frequently in cases with lesion in the L segment (L vs. UM, *P* = 0.032), large resected specimen (58.3 ± 29.5 vs. 39.9 ± 14.4 mm, *P* < 0.001), and large tumor size (25.3 ± 19.9 vs. 17.0 ± 10.6 mm, *P* = 0.011) (Table 4). On the other hand, evaluation of the risk factors in the delayed bleeding subgroup revealed younger age (64.9 ± 12.7 vs. 71.6 ± 8.6 years, *P* = 0.013) and concomitant renal disease (*P* = 0.011) as the risk factors (Table 5).

**Table 3:** Analysis of risk factors related to post-ESD bleeding

	**Bleeding group (n = 23)**	**Non-bleeding group (n = 237)**	***P *** **value**
Age (year, mean ± SD)	66.4 ± 11.3	71.7 ± 8.6	*0.005*
Gender Man/Woman	16 (69.6%)/7 (30.4%)	328 (75.2%)/108 (24.8%)	0.715 (NS)
Tumor location			
U	1 (4.3%)	74 (17.0%)	0.149 (NS)
M	7 (30.4%)	172 (39.4%)	0.519 (NS)
L	15 (65.2%)	190 (43.6%)	*0.042*
Macroscopic type			
Elevated type/Depressed type	16 (69.6%)/7 (30.4%)	240 (54.9%)/196 (55.0%)	0.250 (NS)
Size of resected specimen			
(mm, mean ± SD)	49.1 ± 25.8	39.9 ± 14.4	*0.005*
Tumor size			
(mm, mean ± SD)	21.1 ± 17.2	17.0 ± 10.6	0.087 (NS)
Histopathology			
Adenoma/Carcinoma	3 (13.0%)/20 (87.0%)	49 (11.2%)/387 (88.8%)	0.736 (NS)
Depth of invasion			
M/SM	21 (91.3%)/2 (8.7%)	384 (88.1%)/52 (11.9%)	>0.999 (NS)
Ulcerative findings			
None/present	22 (95.7%)/1 (4.3%)	405 (92.9%)/31 (7.1%)	>0.999 (NS)
Resected style			
One-piece/Piecemeal	21 (91.3%)/2 (8.7%)	382 (87.6%)/54 (12.4%)	>0.999 (NS)
Heart disease	4 (17.4%)	68 (15.6%)	0.770 (NS)
Renal disease	3 (13.0%)	18 (4.1%)	0.081 (NS)
Hepatic disease	4 (17.4%)	41 (9.4%)	0.267 (NS)
Pulmonary disease	4 (17.4%)	32 (7.3%)	0.096 (NS)
Brain disease	3 (13.0%)	42 (9.6%)	0.484 (NS)
Hypertension	12 (52.2%)	197 (45.2%)	0.659 (NS)
Diabetes mellitus	4 (17.4%)	71 (16.3%)	0.778 (NS)
Hyperlipidemia	4 (17.4%)	63 (14.4%)	0.760 (NS)

**Table 4:** Analysis of risk factors related to immediate bleeding after ESD

	**Immediate bleeding group (n = 12)**	**Non-immediate bleeding group (n = 447)**	***P *** **value**
Age (year, mean ± SD)	67.8 ± 9.7	71.5 ± 8.8	0.142 (NS)
Gender Man/Woman	8 (66.7%)/4 (33.3%)	335 (74.9%)/108 (25.1%)	0.710 (NS)
Tumor location			
U	0 (0%)	75 (16.8%)	0.230 (NS)
M	3 (25.0%)	176 (39.4%)	0.382 (NS)
L	9 (75.0%)	196 (43.8%)	*** 0.032***
Macroscopic type			
Elevated type/Depressed type	9 (75.0%)/3 (25.0%)	251 (54.9%)/196 (46.3%)	0.246 (NS)
Size of resected specimen			
(mm, mean ± SD)	58.3 ± 29.5	39.9 ± 14.4	*** 0.005***
Tumor size			
(mm, mean ± SD)	25.3 ± 19.9	17.0 ± 10.6	*** 0.011***
Histopathology			
Adenoma/Carcinoma	2 (16.7%)/10 (83.3%)	71 (15.9%)/376 (84.1%)	>0.999 (NS)
Depth of invasion			
M/SM	11 (91.7%)/1 (8.3%)	412 (92.2%)/35 (7.8%)	>0.999 (NS)
Ulcerative findings			
None/present	12 (100%)/0 (0.0%)	414 (92.6%)/33 (7.4%)	>0.999 (NS)
Resected style			
One-piece/Piecemeal	12 (100%)/0 (0%)	434 (97.1%)/13 (2.9%)	>0.999 (NS)
Heart disease	3 (25.0%)	69 (15.4%)	0.413 (NS)
Renal disease	0 (0.0%)	21 (4.7%)	>0.999 (NS)
Hepatic disease	3 (25.0%)	42 (9.4%)	0.103 (NS)
Pulmonary disease	3 (25.0%)	33 (7.4%)	0.060 (NS)
Brain disease	3 (25.0%)	42 (9.4%)	0.103 (NS)
Hypertension	6 (50.0%)	203 (45.4%)	0.983 (NS)
Diabetes mellitus	1 (8.3%)	74 (16.6%)	0.700 (NS)
Hyperlipidemia	3 (25.0%)	64 (14.3%)	0.396 (NS)

**Table 5:** Anaysis of risk factors related to delayed bleeding after ESD

	**Delayed bleeding group (n = 11)**	**Non-delayed bleeding group (n = 448)**	***P *** **value**
Age (year.mean ± SD)	64.9 ± 12.7	71.6 ± 8.6	*0.013*
Gender Man/Woman	8 (72.7%)/3 (27.3%)	336 (75.0%)/112 (25.0%)	>0.999 (NS)
Tumor location			
U	1 (9.1%)	74 (16.5%)	>0.999 (NS)
M	4 (36.4%)	175 (39.1%)	>0.999 (NS)
L	6 (54.5%)	199 (44.4%)	0.551 (NS)
Macroscopic type			
Elevated type/Depressed type	7 (63.6%)/4 (36.4%)	249 (55.6%)/199 (44.4%)	0.762 (NS)
Size of resected specimen			
(mm, mean ± SD)	39.2 ± 16.1	40.4 ± 15.3	0.800 (NS)
Tumor size			
(mm, mean ± SD)	16.5 ± 12.2	17.3 ± 11.0	0.832 (NS)
Histopathology			
Adenoma/Carcinoma	2 (18.2%)/9 (81.8%)	71 (15.8%)/377 (84.2%)	0.690 (NS)
Depth of invasion			
M/SM	11 (100%)/0 (0.0%)	412 (92.0%)/36 (8.0%)	>0.999 (NS)
Ulcerative findings			
None/present	9 (81.8%)/2 (18.2%)	417 (93.1%)/31 (6.9%)	0.184 (NS)
Resected style			
One-piece/Piecemeal	11 (100%)/0 (0%)	435 (97.1%)/13 (2.9%)	>0.999 (NS)
Heart disease	1 (9.1%)	71 (15.8%)	>0.999 (NS)
Renal disease	3 (27.3%)	18 (4.0%)	*0.011*
Hepatic disease	1 (9.1%)	44 (9.8%)	>0.999 (NS)
Pulmonary disease	1 (9.1%)	35 (7.8%)	0.597 (NS)
Brain disease	0 (0%)	45 (10.0%)	0.611 (NS)
Hypertension	6 (54.5%)	203 (45.3%)	0.763 (NS)
Diabetes mellitus	3 (27.3%)	72 (16.1%)	0.399 (NS)
Hyperlipidemia	1 (9.1%)	66 (14.7%)	>0.999 (NS)

## Discussion

Several studies have reported the usefulness of second-look endoscopy following endoscopic hemostasis to prevent bleeding in patients with hemorrhagic peptic ulcer [7]– [9]. Based on such studies, second-look endoscopy has come to be conventionally performed after ESD as well. Recently, we found reports, albeit only a few, of the benefit of second-look endoscopy following ESD. Kim et al. support the performance of second-look endoscopy following ESD [23], whereas Goto et al. and Ryu et al. reported that second-look endoscopy was not effective for preventing post-ESD bleeding [24,25]. In our study, no difference was observed in the postoperative bleeding rate between the groups that did and did not undergo second-look endoscopy. Consistent with the reports of Goto et al. and Ryu et al., our results suggested that second-look endoscopy following ESD did not reduce the risk of postoperative bleeding. One of the possible reasons is the effect of the gastric pH. Control of bleeding is known to be difficult in the presence of a low gastric pH [13,26]. As the gastric pH is low in patients undergoing endoscopic treatment for peptic ulcer, rebleeding is prone to occur in these patients, and second-look endoscopy is considered to be useful. In contrast, the gastric pH is high at the time of ESD due to the PPI therapy initiated from the previous day, and the risk of rebleeding is lower; therefore, second-look endoscopy may not be required. As the base of a peptic ulcer in the active phase is often covered with a white moss, it is difficult to visually recognize the narrow blood vessels, though thick blood vessels can be visually recognized. Therefore, it is difficult to treat narrow vessels. On the other hand, no white moss is observed in ulcers at the end of the ESD and the narrow vessels can be more clearly recognized, and, therefore, can also be treated. It has been reported that treatment of the visualized blood vessels using hemostatic forceps or a clip at the end of ESD is very useful for reducing post-ESD bleeding [19,20]. Furthermore, Tsuji et al. reported that postoperative bleeding is more common at the margin than at the center of the ulcer base [27]. At our hospital, we treat the visible blood vessels focusing on the ulcer margin in all patients at the end of ESD. As it is possible to treat more blood vessels at the end of ESD than at the time of endoscopic treatment of peptic ulcers, performance of second-look endoscopy following ESD may not have any influence on the incidence of postoperative bleeding.

There are many reports of the risk factors for post-ESD bleeding, including flat or depressed-type lesions, lesions in the L segment, large resected specimens, long operative time, beginner surgeons, patients under maintenance dialysis, and oral intake of antiplatelet agents [5,24,28,29]. Among these, lesions in the L segment and large resected specimens have been reported from multiple researches. In our study, postoperative bleeding was more common in patients with a large resected specimen, lesion in the L segment, and young patients. As bleeding within 24 hours of the ESD procedure accounts for approximately half of all cases of postoperative bleeding, the risk factors for bleeding within 24 hours of ESD (immediate bleeding) and those for bleeding occurring later (delayed bleeding) were investigated. This is the first report of investigation of patients with post-ESD bleeding in detail over time. In this study, immediate bleeding occurred significantly more frequently in patients with lesions in the L segment, large resected specimens, and large tumor size. On the other hand, delayed bleeding was significantly more common in younger patients and patients with concomitant renal disease. In other words, lesion factors predominantly affected the bleeding risk in the early stage after the ESD and patient factors predominantly affected the bleeding risk in the later stages after the procedure. In the case of large resected specimens, multiple blood vessels are present in the ulcer base, according to their size, increasing the risk of bleeding. Okada et al. reported that the volume of postoperative bleeding was 8.2-fold higher when the resected specimen was larger than 4 cm in diameter [29]. It has been shown that the number and diameter of submucosal arteries in the L segment are less and smaller, respectively, than those in the other gastric segments [30], suggesting that lesions in the L segment of the stomach may be associated with less intraoperative bleeding and therefore less hemostatic intervention. As lesions in the L segment are prone to bile exposure which refluxes into the stomach, and a large amount of local injection solution enters the submucosal layer in this segment, blood vessels that cannot be confirmed immediately after the procedure may be exposed as the volume of the local injection solution decreases. With regard to age, Jang et al. reported that the frequency of ESD-associated bleeding (including intraoperative bleeding) was higher in patients younger than 65 years of age as compared with that in patients who were 65 years of age or older [31]. This may be attributable to the higher post-ESD physical activity as compared to elderly patients undergoing the procedure, and also the greater acid secretion in young people than in the elderly. Patients with renal disease have delayed wound healing as a result of tissue fragility, hypoproteinema, and vascular disorders, as well as more marked aggressive factors such as enhanced acid secretion and increased gastrin levels, and reduced defense factors such as prostaglandins, all of which may be expected to lead to a delay in histological restoration of the ulcer and delayed postoperative bleeding [5,32].

In order to minimize the risk of postoperative bleeding, we administer oral PPIs from the day before the operation and use hemostatic treatment with hemostatic forceps or a clip for exposed vessels at the base of the ulcer immediately after the procedure. In addition to ensuring these measures, we believe that some additional measures would be required in patients with large resected specimens or lesions in the L segment and younger patients, who were found in this study to be more prone to postoperative bleeding. For example, it is necessary to identify blood vessels at the ulcer base after ESD, that are prone to cause postoperative bleeding, using endoscopic Doppler ultrasound or infrared imaging system [33,34], and to use over-the-scope-clip for cerclage of the ESD ulcer and medical adhesives for covering the ulcer [35,36]. As these devices and drugs have been examined in only a small number of patients, further study is desired. Second-look endoscopy may be useful in patients who are prone to develop postoperative bleeding 24 hours or later after ESD, such as young patients and patients with renal disease; therefore, further studies are required.

A limitation of this study was that it was a retrospective single-institution study. Furthermore, the number of patients undergoing second-look endoscopy was lower in the first half of the study period than in the second half of the study period, which may have caused a bias.

## Conclusion

In conclusion, performance of second-look endoscopy within a few days after ESD did not reduce the incidence of postoperative bleeding, and is hence considered to be unnecessary. Postoperative bleeding was more likely to occur within 24 hours of the ESD in patients with large lesions/resected specimens and lesions in the L segment, and more likely to occur 24 hours or later in younger patients and patients with concomitant renal disease. However, the actual benefit of second-look endoscopy must be elucidated in a prospective randomized controlled trial in the future, and the risk factors for postoperative bleeding identified in this study should be evaluated further.

## Competing interests

The authors declare that they have no competing interests.

## Authors’ contributions

FT and NY were involved in the design of this study. NY, TA, HK, MN, CT and KT (Kohei Tsuchida) performed the endoscopic treatment. FT, NY, TA and MN conducted data collection and statistical analysis. The manuscript was written by FT and NY, and KT (Keiichi Tominaga), TS, HM and HH provided advice on the manuscript. All authors read and approved the final manuscript.

## Pre-publication history

The pre-publication history for this paper can be accessed here:

http://www.biomedcentral.com/1471-230X/14/152/prepub
